# Moving online: Experiences and potential benefits of digital dance for older adults and people with Parkinson’s disease

**DOI:** 10.1371/journal.pone.0277645

**Published:** 2022-11-18

**Authors:** Judith Bek, David Leventhal, Michelle Groves, Charlotte Growcott, Ellen Poliakoff

**Affiliations:** 1 School of Psychology, College of Social Sciences and Law, University College Dublin, Dublin Ireland; 2 Division of Psychology Communication and Human Neuroscience, School of Health Sciences, Faculty of Biology Medicine and Health, University of Manchester, Manchester, United Kingdom; 3 Mark Morris Dance Group—Dance for PD, Brooklyn, NY, United States of America; 4 Faculty of Education, Royal Academy of Dance, London, United Kingdom; PLOS (Public Library of Science), UNITED KINGDOM

## Abstract

**Background:**

Dance provides a range of beneficial effects for older adults including individuals with age-related neurological conditions such as Parkinson’s disease (PD). The COVID-19 pandemic accelerated the development of at-home dance programs delivered digitally through live and pre-recorded media, but little is known about how participants may engage with and benefit from these resources.

**Objective:**

This study explored experiences and potential benefits of digital dance participation among healthy older adults and people with PD.

**Methods:**

An online survey consisting of fixed-choice and open questions was designed in collaboration with dance program providers and distributed between June and November 2020.

**Results:**

Healthy older adults (N = 149) and people with PD (N = 178) participating in at-home dance programs reported frequent engagement and a range of benefits. People with PD reported greater levels of motor (e.g., ease of movement, balance) than non-motor (e.g., energy, confidence) outcomes, while healthy older adults reported similar numbers of motor and non-motor outcomes. Positive outcomes were associated with the use of movement imagery during dance in both groups, while singing was associated with benefits in people with PD and vocalising was associated with benefits in older adults. At-home dance resources were found to offer convenience and flexibility, but participants missed the interaction, support, and routine provided by in-person classes. The majority expressed a preference to continue with both digital and in-person participation in the future. Qualitative analysis of participants’ comments further revealed that digital participation could help to maintain connection and well-being, as well as identifying further considerations for improving accessibility and facilitating digital engagement.

**Conclusions:**

At-home dance appears to be accessible, engaging, and potentially beneficial for older adults and people with PD, although barriers to participation should be addressed. Digital resources will be increasingly important to enable cost-effective, large-scale provision of home-based therapeutic activities.

## Introduction

The prevalence and economic and societal burden of neurodegenerative conditions such as Parkinson’s disease (PD) is increasing with the ageing population [1]. High levels of physical inactivity among older adults [2] are associated with a wide range of poor health outcomes, including non-communicable diseases, impaired activities of daily living, falls, cognitive decline, and mental health conditions [3]. Inactivity is further exacerbated in people with PD, who are estimated to be around one-third less physically active in comparison to the general population [4]. There is a critical need to identify and develop sustainable methods to increase levels of activity, independence, and quality of life in older adults and those with conditions such as PD.

The contribution of creative approaches to health promotion and rehabilitation is increasingly recognised, as indicated by a recent review of the evidence on arts and health by the World Health Organization [5]. Dance has received particular attention from health professionals and researchers as a form of activity that may provide an engaging and beneficial option for older people, and quantitative and qualitative studies have demonstrated a range of promising effects. Systematic reviews and meta-analyses have documented evidence that dance may reduce the risk of falls and increase strength, balance and mobility in older adults [6–8], as well as improving non-motor outcomes including cognitive function [9–11] and psychosocial well-being [10, 11]. The potential motor and non-motor benefits of dance for people living with ageing-related neurological conditions, particularly PD, as well as others such as stroke and dementia, have also been investigated. Numerous reviews of the research into dance for people with PD have highlighted evidence for improvements in motor symptoms, functional mobility, cognition, and quality of life [12–18], while qualitative evidence also indicates further non-motor effects such as increased social participation and confidence [19–21]. In particular, there is clear evidence for motor improvements following dance participation in people with PD. For example, a randomised controlled trial comparing Irish dancing with standard physiotherapy exercises found greater improvements in balance, freezing of gait, and overall motor symptom severity in the dance group [22]. Moreover, a recent study found that long-term dance participation may slow the progression of motor symptoms in people with mild PD [23]. There is also evidence that dance can improve physical, cognitive, and psychological outcomes in other neurological conditions including stroke [9, 24] and dementia [9, 25].

Although dance incorporates physical exercise, the complexity of dance as a motor, cognitive, emotional and social activity likely contributes to its positive outcomes [26, 27], and dance has been associated with enhanced neural plasticity when compared with repetitive exercise in older adults [28]. Importantly, older adults and people with PD show high levels of adherence to dance [8, 14, 27], indicating its potential as an effective and sustainable approach.

The restrictions in social contact and participation imposed in response to the COVID-19 pandemic significantly impacted older people, many of whom faced the highest levels of isolation because of the age-related risk factors associated with the virus [29, 30]. In these circumstances, the importance of telehealth [31–33] and digital alternatives such as apps and online programs for maintaining communication and activity [33, 34] was brought to the forefront. Older people are increasingly confident in using digital technology, and studies during the pandemic reported that digital resources were used by older adults to maintain social connections [35] and by people with PD to engage in physical activity [32, 36]. Nonetheless, the level of technology use among older adults is likely still lower than in other age groups [37], and so their use of online social and health resources may be more limited.

In response to the suspension of in-person group activities during the pandemic, providers of community dance programs (including initiatives designed specifically for older adults and people with PD) transitioned to delivering classes online. Although a small number of previous studies have investigated home-based dance for older adults [38] and people with PD [22, 39], these typically involved offline resources provided alongside in-person dance classes or within a videogame, so the effects of online and group-based digital dance programs are unclear. Considering the potential barriers relating to the use of technology, and the differences between in-person classes and online participation (such as levels of social contact and instructor feedback), the present study used an online survey to investigate users’ experiences and perceived benefits of digital dance resources.

The survey explored access to and engagement with home-based dance programs among older adults, as well as the potential outcomes of these programs across motor and non-motor domains. For individuals with neurological conditions like PD, there may be additional barriers or challenges to participation, such as reduced motivation [40] or concerns about safety [41], or they may be less able to experience the benefits of dance without the support and guidance provided within group classes. The present study therefore also sought to identify any differences in the types of benefits experienced by neurologically healthy older adults and those with PD, as well as factors that may influence outcomes for each user group. Initial findings of the survey based on data collected from people with PD [42] indicated that home-based dance programs were accessed with high levels of engagement and revealed a range of potential motor and non-motor benefits. This initial analysis also found that perceived benefits were greater among participants engaging in strategies such as movement imagery during dance classes. The present study further examines such mechanisms of engagement and their relationship with potential benefits, in older adults without neurological conditions and people with PD. Qualitative analysis was also conducted to explore participants’ experiences in greater depth.

## Methods

An online survey was designed in collaboration with providers of community dance programs for older adults and people with PD (Silver Swans®—Royal Academy of Dance; Dance for PD®—Mark Morris Dance Group), with additional input from dance instructors, physiotherapists, and individuals with PD who had previously participated in dance.

The survey (see S1 File) consisted primarily of fixed-choice questions addressing access to and use of digital dance programs, including engagement in cueing strategies during dance (e.g., imagery, vocal and rhythmic cues), perceived benefits, advantages and disadvantages of at-home participation, and views on future participation. Two optional questions were included to allow participants to provide further information if they wished (“*Do you have any suggestions for how at-home dance resources could be improved*?”; “*Is there anything else you would like to tell us about your experience of at-home dance resour*ces?”). The survey was also open to individuals who were interested but not currently participating in dance, who were directed to a subset of questions addressing previous participation, barriers to accessing resources, and interest in future participation.

The survey was administered using SelectSurvey.net (v4·033·002; ClassApps, Overland Park, KS, USA). Invitations to participate in the survey were distributed between June and November 2020 through email newsletters of dance organisations and Parkinson’s UK, as well as dance practitioners known to the research group, older adult groups/networks (e.g., Age of Creativity; University of the Third Age), research volunteer lists, and social media. The study was approved by the University of Manchester Research Ethics Committee and participants provided informed consent via an online form, completion of which was required in order to access the survey.

### Participants

Valid survey responses were collected from 466 individuals, including 276 people living with PD and 16 with other neurological conditions, as well as 5 who did not report whether or not they had a neurological condition. Respondents were aged between 43 and 89 years, and the majority were based in the UK (72.1%) or USA (20.4%).

### Data analysis

Since only 3.4% of participants reported a neurological condition other than PD, and a further 1.1% did not report whether or not they had a neurological condition, the present analysis focuses on older adults without any neurological condition (OA) and people with PD.

Responses to fixed-choice questions were summarised as percentages and are reported descriptively in the following section. Statistical analyses were conducted to further examine outcomes of participation in the two main respondent groups (OA and PD). Twelve items from a checklist of potential outcomes of dance participation were categorised as either motor or non-motor outcomes (6 items per category), which were then compared using a group (OA, PD) x type (motor, non-motor) ANOVA. Given that the majority of respondents were from the UK or USA, the numbers of perceived benefits reported by people with PD in the two countries (UK N = 97; USA N = 69) were compared using an independent t-test. This analysis was not conducted for the OA group because the data included a much larger number of OA respondents from the UK (N = 116) than the USA (N = 20). To explore how the use of particular strategies within at-home dance participation might be associated with reported benefits, linear mixed effects modelling was conducted in R [43] using the package lme4 [44]. This approach enables the effects of fixed factors (predictors) to be analysed while adjusting for random effects arising from individual variation between participants. Responses to the two open-ended survey questions were analysed using an inductive/data-driven thematic approach [45]. Two of the authors (CG and JB) independently coded the responses, using Microsoft Excel and NVivo to identify and categorise codes. Candidate themes were generated, which were then discussed further with a third author (MG) before being finalised.

## Results

### Experiences and perceptions of accessing and using at-home dance resources

The characteristics of all respondents (including OA and PD, as well as individuals reporting other neurological conditions or not reporting any neurological conditions) currently using at-home dance programs (N = 348) are summarised in Table 1. The length and frequency of participation in at-home dance programs was similar between OA and PD respondents: the majority had been participating for less than 12 months and most were currently participating at least once a week. Most of the OA respondents (95.3%) and 78.7% of the PD respondents had previously attended in-person classes.

**Table 1:** Characteristics of participants currently using home-based resources, summarised for all users of at-home dance programs (including those with and without any neurological conditions) and separately for older adults without a neurological condition (OA) and individuals with Parkinson’s disease (PD).

	*All users (N = 348)*	*OA (N = 149)*	*PD (N = 178)*
Age (years: M; range)	66.9; 43–88	66.0; 50–89	69.5; 47–88
Gender (% female; male)	83.2; 14.5	94.6; 4.7	75.8; 24.2
Previous in-person dance participation (% yes)	85.2	95.3	78.7
Frequency of at-home practice (%):			
Less than once weekly	5.7	6.7	5.1
Once weekly	30.5	32.2	29.2
Twice weekly	31.6	36.2	28.1
More than twice weekly	29.6	24.2	36.5
Length of participation (%):			
0–3 months	37.3	48.3	29.2
3–6 months	26.8	28.2	26.4
6–12 months	13.4	8.1	18.5
>12 months	15.1	12.8	16.9
Use of other resources for home-based activities (% yes)	64.7	53.7	77.5
Use of online platforms/media for social interaction (% yes)	74.4	83.2	73.6

Table 2 summarises the responses to questions exploring access to and use of digital resources, barriers to participation, preferences in the selection and use of resources, and considerations for future provision. The results are summarised separately for OA and PD respondents in addition to the overall results from all respondents using digital dance resources.

**Table 2:** Survey responses summarised as percentages of participants endorsing each item presented separately for all users of at-home dance programs, older adults (OA), and individuals with Parkinson’s disease (PD). The most frequent responses to each item are indicated in bold for each group.

	*All users*	*OA*	*PD*
*Media type used*:			
**Live-streamed class**	**63.5**	**63.1**	66.3
Interactive online class (e.g., Zoom)	49.6	55.0	47.2
**Pre-recorded online class**	62.7	57.7	**69.1**
DVD	17.1	1.3	28.7
Other (e.g., printed instruction)	1.1	0.7	1.1
*Preferred media*:			
**Live**	**64.0**	**45.8**	**64.5**
Pre-recorded	19.8	15.0	18.7
Both live and pre-recorded	21.8	28.0	11.2
No preference	3.6	1.9	3.7
*Factors influencing choice of resources*:			
**Dance style (e.g., ballet, modern, mixed)**	**68.4**	**79.9**	**60.1**
Familiar program/instructor	57.3	61.7	55.6
Free classes only	27.1	23.5	32.0
Low cost	35.6	31.5	39.9
Scheduled classes	53.0	53.7	53.4
Opportunities for social connection	29.1	27.5	30.9
Reputation/brand	35.0	35.6	34.3
Recommendation	35.3	34.2	37.6
Difficulty level	36.8	32.9	41.6
Type of media/platform	40.2	43.6	37.6
*Difficulties in accessing or using resources*:			
Connectivity/network problems	35.9	42.2	29.2
Setting up or using software	7.7	8.1	7.3
Image quality	8.8	10.7	6.7
Sound quality	20.5	27.5	15.2
**No problems**	**50.4**	**42.3**	**61.8**
*Aspects missed from in-person participation*:			
Interaction with the instructor	69.5	77.2	63.5
**Interaction with others**	**77.8**	**89.3**	**70.8**
Support/encouragement	44.7	51.7	38.8
Live music	31.1	30.2	31.5
Social activities before/after class	43.3	47.0	40.5
*Plans for future participation*:			
Attend in-person classes only	18.2	24.2	12.4
Continue with home practice only	9.7	6.0	13.5
**Both attend classes and continue with home practice**	**68.4**	**69.1**	**70.8**
Neither	0.3	0.0	0.6
*Interest in educational resources to supplement practice*:			
Written information	8.9	8.7	9.6
Educational video	11.1	8.7	13.5
**Both written information and video**	**51.6**	**47.0**	**56.2**
Neither	26.5	34.9	19.7
*Interest in trying new technology-based approaches to dance*:			
Practicing dance movements using an app	20.8	22.8	19.1
Virtual reality dance	9.4	8.7	9.0
**Both app and virtual reality**	**44.2**	**37.6**	**51.7**
Neither	23.4	30.2	19.1

Both live and pre-recorded classes were widely used, although the use of DVDs was more common among people with PD, possibly reflecting the availability of at-home DVDs from the Dance for PD® program predating the pandemic. Among those using both live and recorded media, there was a preference for live classes, although this was more evident in the data from participants with PD (64.5%) than OA (45.8%). The most important factor in choosing at-home programs was the style of dance, followed by familiarity and scheduled classes. Half of all participants experienced some technical difficulties when accessing or using resources, most commonly involving connectivity/network problems, although OA respondents reported more difficulties than PD respondents.

**Fig 1:**
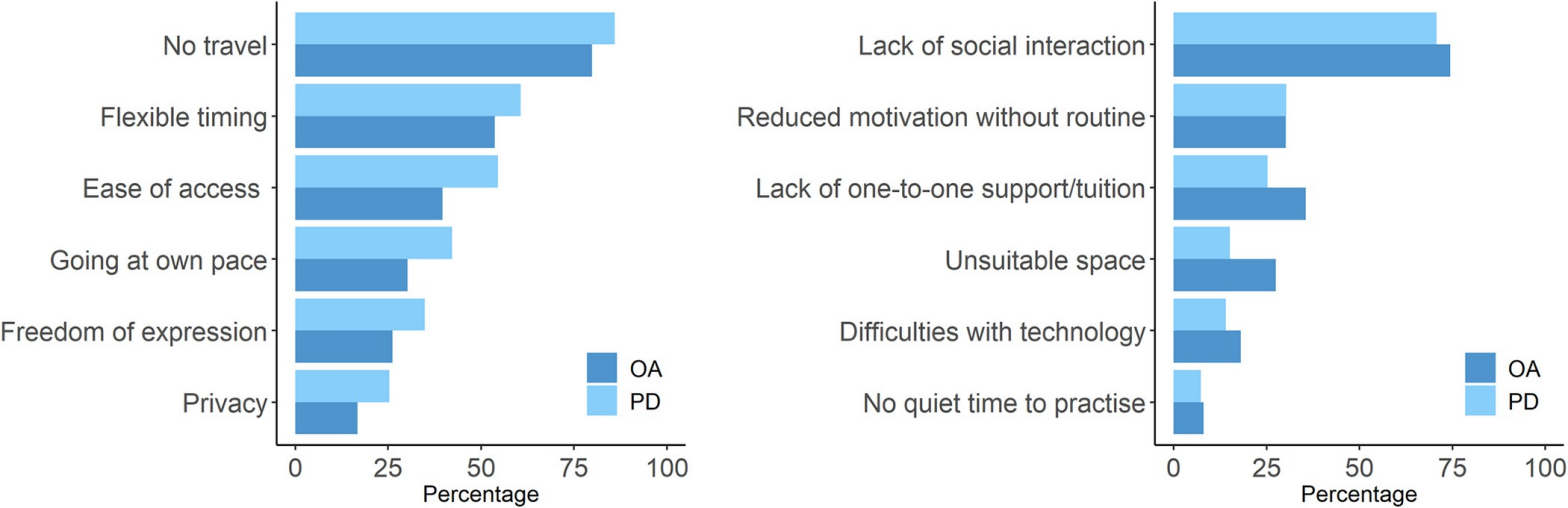
Advantages (left) and disadvantages (right) of at-home dance participation reported by older adults (OA) and people with PD.

The reported advantages and disadvantages of home-based dance participation are illustrated in Fig 1, which shows a broadly similar pattern of results between groups, although OA respondents tended to report disadvantages more frequently and advantages less frequently than those with PD. In both groups, the most widely reported advantages were not having to travel, flexible timing, and ease of access enabling more frequent participation. The key disadvantages were the lack of social interaction and one-to-one support, and reduced motivation resulting from the loss of routine.

Participants previously attending in-person classes primarily missed the interaction with other participants and instructors, and these aspects were more frequently noted by OA than PD respondents. Nonetheless, the data clearly indicated a desire among both groups to continue participating in at-home digital classes alongside in-person classes when these resumed. The majority of participants were also interested in receiving written and visual resources to supplement their practice, as well as using apps or virtual reality for dance.

### Reported benefits and strategies

**Fig 2:**
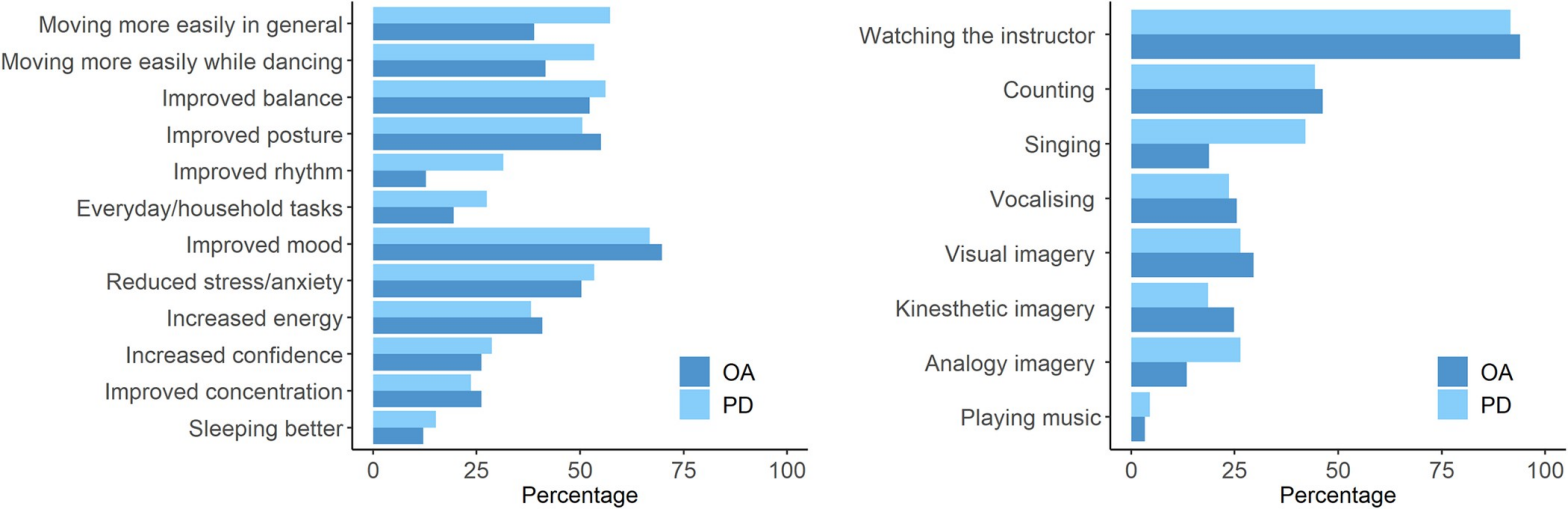
Outcomes of at-home dance (left; percentage of participants reporting effects) and strategies used during dance (right; percentage of participants engaging in each strategy) as reported by older adults (OA) and people with PD.

As shown in Fig 2 (left panel), both OA and PD respondents reported a range of motor benefits (e.g., improved posture and balance, greater ease of movement) and non-motor benefits (e.g., improved mood, reduced stress/anxiety, increased energy). Only 6% of OA respondents and 5.1% of PD respondents did not perceive any benefits of at-home dance participation.

Statistical analysis showed no significant effect of group on the overall number of benefits reported (*F*(1, 312) = 1.89; *p* = .17; *η*^*2*^*G* = .0045), but there was a significant effect of outcome type (*F*(1, 312) = 3.99; *p* = .047; *η*^*2*^*G* = .0033), and a significant group x type interaction (*F*(1,312) = 10.02; *p* = .0017; *η*^*2*^*G* = .0082). Paired t-tests revealed that people with PD reported significantly more motor than non-motor benefits (mean difference = .53; *t*(171) = 4.16; *p* < .0001; *d* = .32), while OA respondents reported similar numbers of motor and non-motor benefits (mean difference = .12; *t*(141) = .73; *p* = .47; *d* = .061). Comparison of the outcomes reported by people with PD in the UK versus USA revealed a significant difference (t(129) = 2.37; p = .0019; d = .38), with participants in the USA reporting more benefits (M = 5.94; SD = 3.38) than those in the UK (M = 4.74; SD = 2.85).

Fig 2 (right panel) shows the reported use of strategies and cues during at-home dance participation. The relationship between strategy use and perceived benefits within each group (PD and OA) was explored using linear mixed-effects modelling. A baseline model included the intercept for participants as a random effect and outcome type (motor vs. non-motor) as a fixed factor. To determine the potential influence of specific aspects of engagement, the following strategies and their interactions with outcome type were entered into the model for each group: counting, vocalising, singing, visual imagery, kinesthetic imagery, and analogy/metaphor imagery. Two further items, “watching the instructor closely” and “playing my own music in the background”, were omitted from the analysis due to the respectively very high and low percentages of participants endorsing these. Strategies were added sequentially into the models for each group to identify which factors contributed significantly to the reported benefits. Likelihood ratio tests were used to compare each model with the previous one. Based on these tests, strategies significantly improving the fit of the model were retained and those not significantly contributing to the model were removed.

For OA respondents, vocalising contributed significantly compared to the baseline model (*χ*^*2*^(2) = 7.30; *p* = .026) and visual imagery further improved the prediction of outcomes (*χ*^2^(2) = 6.04; *p* = .048). The final model showed a significant main effect of visual imagery (*b* = .78, SE  = 0.32, *t*(243.4)   =   2.43; *p*  =  .016) and a significant interaction between vocalising and outcome type (*b* =  .98, *SE*  =  0.37, *t*(142)   =   2.65; *p*  =  .009), indicating a specific positive association with non-motor benefits.

For PD respondents, the baseline model was improved by the addition of singing (*χ*^2^(2) = 9.0; *p* = .011), and both visual imagery (*χ*^2^(2) = 9.91; *p* = .007) and analogy/metaphor imagery (*χ*^2^(2) = 13.95; *p* < .001) contributed further to the model. In the final model there were significant main effects of singing (*b*  = .53, *SE * =  0.26, *t*(272.2)   = 2.04; *p * =  .042) and visual imagery (*b*  = .71, *SE * =  0.29, *t*(272.2)   = 2.44; *p * =  .015), as well as a significant interaction between analogy/metaphor imagery and outcome type (*b*  = - 0.633, *SE * =  0.29, *t*(172)   =  -2.17; *p * =  .031), indicating a stronger association with motor than non-motor benefits.

### Qualitative analysis

Thematic analysis did not indicate any clear differentiation between OA and PD respondents in the content of the open comments, so themes were generated across the combined OA and PD data. The themes are summarised in Table 3 with example quotes. Full details of the themes are provided in the S2 File.

**Table 3:** Themes generated from open comments of older adults (OA) and individuals with PD (PD).

*Theme 1*. *Maintaining connection and well-being*. Participants missed the social connection of in-person classes, but a sense of community could nonetheless be found through connecting with others online: *“Nothing replaces actually being in the dance studio with a teacher and other dancers” [OA]* *“Although I … am enjoying them at present*, *I still prefer real life classes*, *with all their interaction” [PD]* *“You feel connected to the rest of the group even if it’s done remotely” [OA]* *“…feel I am part of something even though no-one else is there*!*” [PD]* Being able to access at-home dance programs supported participants in maintaining their physical and mental health during COVID-19 restrictions: *“I feel as though I have met new friends*. *What could have been better during this period of lockdown and global pandemic*. *I do think it is of paramount importance that people of my age continue to do the things they love*, *to keep them fit and healthy and in a good place mentally*.*” [OA]* *“I am really enjoying dance at home and feel much better physically and mentally after a class*. *It is especially beneficial at this time*, *when there are so many restrictions*.*” [PD]*
*Theme 2*. *Advantages and opportunities offered by online participation*. This theme highlighted advantages of at-home participation, such as the convenience of not having to travel, the capacity to dance more frequently, and the provision of a safe space for movement and expression: *“Please continue after COVID-19*. *No traveling to class huge benefit*!!!*” [PD]* *“The online experience of classes enables me to choose (having tried several different ones) which one I feel I am able to do that day and at a time convenient to me*.*” [OA]* *“Allows people who are physically restricted to move freely and use their creativity and imagination without being judged” [PD]* The desire to continue with online classes was also evident: *“I have enjoyed the dance experiences and intend to continue in class and online in the future” [OA]* *“I also believe it would continue to be useful in the future for those who are unable to travel to ordinary live classes*, *and for those who are keen to dance as much as possible” [PD]* Online participation led to new experiences and opportunities for some participants, such as the possibility of joining classes in remote locations and meeting new people online, or trying new dance styles: *“I have also enjoyed some online classes found on YouTube which were taught in different ways” [OA]* *“I also like meeting dancers from all over the world*. *If not virtual*, *I would not be able to attend*. *A gift*!*” [PD]* *“…the interaction with people from across the world was fascinating and motivating” [PD]* It was also noted that the learning of new technology skills had been accelerated during the pandemic: *“It has made me feel more confident using video and computer technology” [OA]*
*Theme 3*. *Effectiveness of engagement*, *teaching*, *and learning*. This theme encapsulated both positive and negative aspects of at-home classes. For example, it was noted that it could be difficult to maintain motivation, although scheduled classes encouraged regular participation: *“I find having a regular time slot enables me to plan for it” [PD]* *“It was good to be able to keep the connection with the weekly class as it helps motivate a structure in the day at a specific time*.*” [OA]* It was also suggested that consistency and familiarity of the group and the instructor were important: *“Teachers should maybe keep a class small and there should be a registration so you are in an actual group like a real class without so much come and go of the students” [OA]* *“I like the contact with my regular teacher and understand her way of teaching” [OA]* While the above points could apply equally to in-person and digital classes, other feedback indicated aspects more specific to the home-based context. For example, clear visibility of the movements was noted to be important to facilitate learning: *“Demonstrations of the steps using more than one camera so we can see different angles” [OA]* *“One thing we really value in the at-home dance resources is when instructors are very visible and both describing and doing the movements when showing us a new segment” [PD]* It was also noted that the home environment could be restrictive or unsuitably equipped for dance: *“I find space restricts me—I like to really MOVE and travel while dancing” [PD]* Issues concerning accessibility were highlighted, such as the need to improve technical knowledge and the availability of guidance on how to find suitable resources: *“Training for both instructors and users on how best to use videoconferencing” [PD]* *“Providing a directory or central portal where resources*, *classes etc*. *could be located” [OA]* The need to support participation across different locations, languages, and cultures was suggested: *“More classes in other languages… I often think about all the other people that may not participate because of language barriers*. *In general too*, *I think the at-home resources need to increase advertising for racial and ethnic minorities because it’s a very homogeneous group that participates*.*” [PD]*
*Theme 4*: *Different preferences*. It was evident that preferences varied between participants, such as in the format of digital classes: *“The interactive sessions are great fun*, *the pre-recorded sessions I have greater difficulty*. *I like both” [OA]* *“I wish there were more opportunities to access more than one weekly interactive class” [PD]* *“I would like to be able to access classes in a library*, *to be able to repeat them if wished” [PD]* There were also different preferences for having classes provided as complete sessions or in shorter segments to aid learning: *“I would like a continuous 30–40 minute workout so I don’t need to click on each exercise” [OA]* *“…it would be ideal to have starter videos that break down the steps” [PD]*

## Discussion

The present study provides initial evidence that dance programs delivered through digital media are accessible and engaging for older adults and people with PD, and may provide a range of motor and non-motor benefits similar to those reported from in-person participation [8, 9, 14–18, 26, 46]. This is further supported by other recent evidence from small-scale studies indicating the safety, feasibility, and reported benefits of online dance for people with PD ([47, 48]; for review, see [16]) and findings that virtual PD-specific exercise was well-received and provided motor and non-motor benefits [49].

Although the overall number of perceived benefits of at-home dance participation was similar between OA and PD respondents, people with PD reported a greater number of motor effects, including improvements in posture, balance, and ease of movement. This difference in perceived outcomes between groups may reflect the specific features of the programs used (e.g., PD-specific classes), or different goals and objectives for participating in dance. The latter suggestion is supported by previous qualitative research indicating that addressing motor symptoms is a priority outcome of dance among people with PD [19]. It is also possible that people with PD are simply more aware of their movement difficulties and thus also more aware of any improvements, but differences in outcomes between groups should be examined with quantitative measures in future research. For many older adults without a long-term health condition, dance may be enjoyed primarily as a social leisure activity, with a reduced focus on physical benefits. Consistent with this suggestion, the present study found a greater emphasis on the importance of social interaction in dance among older adults without any neurological conditions.

Analysis of the use of cueing strategies in dance revealed further insights into potential mechanisms of the benefits of digital participation. In both OA and PD respondents, the use of visual imagery was associated with the overall number of benefits reported, while there were differences between the groups for other strategies. Among OA respondents, vocalising the movements was associated with non-motor benefits, while for PD respondents singing was associated with overall benefits and analogy/metaphor imagery was associated with the number of motor benefits. Our previously reported initial analysis of the data from respondents with PD [42] associated imagery and singing with overall benefits, while the further breakdown by outcome domains in the present study indicates that different forms of imagery may be differentially associated with outcomes of dance.

A better understanding of the mechanisms involved in dance participation could help to enhance its beneficial effects. Further to the more commonly recognised elements of dance such as physical activity, rhythm, and social interaction, the present findings clearly indicate the potential value of imagery as a motor-cognitive strategy that may boost positive outcomes for older adults with and without PD. In particular, the use of narrative themes and music [21, 50] could support engagement in the analogy/metaphor-type imagery that often features in dance for people with PD and older adults [51, 52]. Additionally, most of the participants in the present study reported attending closely to the instructor’s movements during their dance practice, and the qualitative data further indicated the importance of clear visual demonstration. As discussed in a recent review [26], movement imagery is widely used in dance, alongside observation and imitation of movements [26, 53]. These motor-cognitive processes are known to activate brain networks overlapping with those involved in motor preparation and execution [54], and have shown therapeutic effects in people with PD (e.g., [55]; for review see [56–58]).

The present findings also suggest that other strategies such as vocalising and singing may contribute to the positive effects of dance. Previous research has indicated that singing may help to provide a rhythmic cue for movement in people with PD [59] and can improve quality of life and reduce anxiety [60, 61]. Researchers have also begun to investigate online delivery of singing therapy for people with PD [62]. The relationship between singing and dancing as a potential combined therapeutic approach should therefore be explored further. The importance of being able to connect with others through dance was clear from both quantitative and qualitative data in the present study, and is consistent with previous reports [21, 63, 64]. While digital participation can only partially replicate the social environment of in-person dance, live online classes in particular could help to maintain a sense of community. The continuation of dance programs, albeit in the virtual domain, was also noted as providing a valuable means of maintaining physical and mental health in times of isolation and anxiety during lockdown. Moreover, the qualitative data revealed that digital dance programs could have some unique advantages, as participants reported their appreciation of opportunities such as discovering new classes or different dance styles, connecting with others in distant locations, and the accelerated learning of digital technology skills. This digital mode of participation also provided a safe space for individuals to practice dance without feeling self-conscious and could help to maintain confidence. There was a desire among respondents to continue participating in online dance alongside in-person classes (as also noted in a recent study of online exercise classes for PD [49]), with an appreciation of the convenience, variety, and frequency of participation offered by digital resources.

At-home programs have clear potential to broaden access to dance and other activities, particularly for those who have difficulty travelling to classes and those in rural communities without in-person provision. Indeed, home-based activity programs have previously shown increased levels of adherence among older adults compared with travelling to attend sessions [65]. Nonetheless, the uptake of online dance programs is also dependent on the availability of, and ability to engage with, the necessary technology. While around half of respondents in the present study had experienced some technical problems, these mostly involved issues with connectivity, rather than lacking knowledge of or access to digital technology. However, the responses of individuals not currently using at-home programs also indicated that many may be unaware of the availability of resources, or may need support to find and access the available options. Moreover, the nature of the present study means that the results only reflect the experiences of those who already have a certain level of digital engagement. Participants’ comments also highlighted the need to increase accessibility of live online classes, by catering to different time zones and more diverse sections of the community such as different language groups, although some program providers are already working to address this [66].

Variation in preferences among participants indicates that providing alternative options, such as different levels of intensity or difficulty, or different dance styles, may increase motivation and engagement. This is consistent with qualitative studies indicating the need for appropriate challenge in dance for people with PD [19, 21]. High-quality instruction, and the experience of instructors in working with particular groups, was noted to be important (see also [33]). The availability of regular scheduled classes was also suggested to help in providing structure and motivation.

Respondents expressed an interest in receiving educational resources to supplement their dance practice. Given the preliminary evidence that the use of strategies such as imagery, singing, and vocalising might enhance outcomes, these could be highlighted within educational materials. There was also interest in trying new technologies for dance participation. Dance exergames have been associated with physical, cognitive and social benefits in older adults [38], and virtual reality based dance has been found to improve balance, daily activities, and mood in PD [67]. There is potential for these technologies to further enhance the at-home dance experience for older people and those with PD. For example, the use of immersive media could facilitate engagement by enabling participants to view the instructors from different angles or focus in on particular movements, thereby supporting motor-cognitive processing.

It should be noted that the findings of the present study may not fully capture the diversity of experiences of older adults using at-home dance programs and resources. As noted above, the nature of the online survey method means that only those with sufficient ability and engagement with digital technology would be reached, and others using offline resources such as DVDs may be less well represented by the results of the survey. In addition, the survey was advertised through groups and networks for older adults and people with PD (including the Silver Swans and Dance for PD networks), but other neurological conditions (e.g., stroke, dementia, and multiple sclerosis) were not specifically targeted. It is also important to acknowledge the limitations of self-report data and the potential influence of fixed-choice responses. For example, in the present survey, participants may have felt obliged to report some benefits or strategies rather than none, or may have over-reported benefits or advantages of their dance practice. Finally, although the survey was shared internationally, the majority of respondents were based in the UK or USA. An exploratory analysis revealed that people with PD in the USA reported a higher number of overall benefits compared to those in the UK. Speculatively, this might reflect differences in the specific programs accessed and/or cultural influences on self-reported outcomes, such as higher levels of optimism among people with PD in the USA. Further research should thus investigate potential cultural differences in the use and outcomes of home-based dance resources.

**Fig 3:**
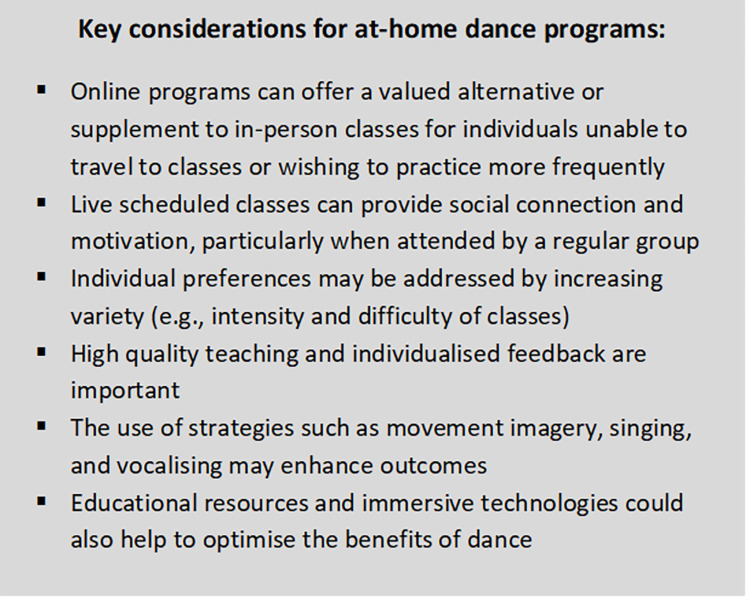
Key considerations for the design of at-home dance programs as indicated by quantitative and qualitative data from the survey.

Based on the present findings, key considerations for the further development of at-home dance programs are summarised in Fig 3. While the provision of digital resources for home-based activities was accelerated as a result of restrictions during the COVID-19 pandemic, online classes are already being integrated into ongoing programming by dance organisations [66], and their use and importance will likely continue to grow as older people’s proficiency in using digital technology increases. Moreover, although digital programs cannot entirely replace in-person activities, they can offer widely available resources to support health and well-being, with the possibility of providing benefits to individuals across geographic and cultural landscapes.

## Conclusion

The present findings indicate that, similar to in-person programs, home-based dance participation can be beneficial for older adults and people with PD, having positive outcomes in both motor and non-motor domains. Additionally, the use of imagery and vocal/rhythmic strategies could enhance the effects of dance participation. Further work is needed to address barriers to participation and to identify longer-term benefits of home-based dance programs, incorporating both quantitative and qualitative outcome measures. Feedback from participants and instructors will also be a valuable tool in the ongoing development of accessible and engaging dance programs.

## Supporting information

S1 File Survey questions. (PDF)

S2 File Themes generated from comments of older adults (OA) and individuals with PD (PD) in response to open survey questions. (PDF)

## References

[1] Dorsey RE, Elbaz A, Nichols E, Abd-Allah F, Abdelalim A, Adsuar JC, et al. Global, regional, and national burden of Parkinson’s disease, 1990–2016: a systematic analysis for the Global Burden of Disease Study 2016. The Lancet Neurology. 2018;17: 939–953. doi: 10.1016/S1474-4422(18)30295-3 30287051 PMC6191528

[2] Gomes M, Figueiredo D, Teixeira L, Poveda V, Paúl C, Santos-Silva A, et al. Physical inactivity among older adults across Europe based on the SHARE database. Age and Ageing. 2017;46: 71–77. doi: 10.1093/ageing/afw165 28181637 PMC6402309

[3] Cunningham C, O’Sullivan R, Caserotti P, Tully MA. Consequences of physical inactivity in older adults: A systematic review of reviews and meta-analyses. Scandinavian Journal of Medicine and Science in Sports. Blackwell Munksgaard; 2020. pp. 816–827. doi: 10.1111/sms.13616 32020713

[4] Nimwegen M Van Speelman AD, Hofman-Van Rossum EJM, Overeem S, Deeg DJH, Borm GF, et al. Physical inactivity in Parkinson’s disease. Journal of Neurology. 2011. doi: 10.1007/s00415-011-6097-7 21614433 PMC3225631

[5] Fancourt D, Finn S. What is the evidence on the role of the arts in improving health and well-being? A scoping review. WHO Regional Office Europe. Available online at: http://www.euro.who.int/en/publications/abstracts/what-is-the-evidence-on-the-role-of-the-arts-in-improving-health-and-well-being-a-scoping-review-2019. 32091683

[6] Fernández-Argüelles EL, Rodríguez-Mansilla J, Antunez LE, Garrido-Ardila EM, Muñoz RP. Effects of dancing on the risk of falling related factors of healthy older adults: A systematic review. Archives of Gerontology and Geriatrics. Elsevier Ireland Ltd; 2015. pp. 1–8. doi: 10.1016/j.archger.2014.10.003 25456888

[7] Mattle M, Chocano-Bedoya PO, Fischbacher M, Meyer U, Abderhalden LA, Lang W, et al. Association of Dance-Based Mind-Motor Activities with Falls and Physical Function among Healthy Older Adults: A Systematic Review and Meta-analysis. JAMA Network Open. 2020;3: 2017688. doi: 10.1001/jamanetworkopen.2020.17688 32975570 PMC7519422

[8] Woei-Ni Hwang P, Braun KL. The effectiveness of dance interventions to improve older adults’ health: A systematic literature review. Alternative Therapies in Health and Medicine. InnoVision Communications; 2015. pp. 64–70. 26393993 PMC5491389

[9] Kshtriya S, Barnstaple R, Rabinovich DB, DeSouza JFX. Dance and Aging: A Critical Review of Findings in Neuroscience. American Journal of Dance Therapy. 2015. doi: 10.1007/s10465-015-9196-7

[10] McNeely ME, Duncan RP, Earhart GM. A comparison of dance interventions in people with Parkinson disease and older adults. Maturitas. 2015;81: 10–16. doi: 10.1016/j.maturitas.2015.02.007 25771040 PMC4497370

[11] Meng X, Li G, Jia Y, Liu Y, Shang B, Liu P, et al. Effects of dance intervention on global cognition, executive function and memory of older adults: a meta-analysis and systematic review. Aging Clinical and Experimental Research. Springer; 2020. pp. 7–19. doi: 10.1007/s40520-019-01159-w 30982217

[12] dos Santos Delabary M, Komeroski IG, Monteiro EP, Costa RR, Haas AN. Effects of dance practice on functional mobility, motor symptoms and quality of life in people with Parkinson’s disease: a systematic review with meta-analysis. Aging Clinical and Experimental Research. Springer International Publishing; 2018. pp. 727–735. doi: 10.1007/s40520-017-0836-2 28980176

[13] Kalyani HHN, Sullivan K, Moyle G, Brauer S, Jeffrey ER, Roeder L, et al. Effects of Dance on Gait, Cognition, and Dual-Tasking in Parkinson’s Disease: A Systematic Review and Meta-Analysis. Journal of Parkinson’s Disease. 2019. doi: 10.3233/JPD-181516 30958312

[14] Sharp K, Hewitt J. Dance as an intervention for people with Parkinson’s disease: A systematic review and meta-analysis. Neuroscience and Biobehavioral Reviews. 2014;47: 445–456. doi: 10.1016/j.neubiorev.2014.09.009 25268548

[15] Shanahan. Dance for People With Parkinson Disease: What Is the Evidence Telling Us? (vol 96, pg 141, 2015). Archives of Physical Medicine and Rehabilitation. 2015;96: 1931–1931. doi: 10.1016/j.apmr.2015.08.420 25223491

[16] Emmanouilidis S, Hackney ME, Slade SC, Heng H, Jazayeri D, Morris ME. Dance Is an Accessible Physical Activity for People with Parkinson’s Disease. Parkinson’s Disease. 2021;2021: e7516504. doi: 10.1155/2021/7516504 34721836 PMC8556098

[17] Wang L, Sun C, Wang Y, Zhan T, Yuan J, Niu C-Y, et al. Effects of dance therapy on non-motor symptoms in patients with Parkinson’s disease: a systematic review and meta-analysis. Aging Clin Exp Res. 2022;34: 1201–1208. doi: 10.1007/s40520-021-02030-7 35091970

[18] Hasan S, alshafie S, Hasabo EA, Saleh M, Elnaiem W, Qasem A, et al. Efficacy of dance for Parkinson’s disease: a pooled analysis of 372 patients. Journal of Neurology. 2022;269. doi: 10.1007/s00415-021-10589-4 33966112

[19] Rocha PA, Slade SC, McClelland J, Morris ME. Dance is more than therapy: Qualitative analysis on therapeutic dancing classes for Parkinson’s. Complementary Therapies in Medicine. 2017;34: 1–9. doi: 10.1016/j.ctim.2017.07.006 28917359

[20] Zafar M, Bozzorg A, Hackney ME. Adapted Tango improves aspects of participation in older adults versus individuals with Parkinson’s disease. Disability and Rehabilitation. 2017;39: 2294–2301. doi: 10.1080/09638288.2016.1226405 27767375

[21] Houston S, McGill A. A mixed-methods study into ballet for people living with Parkinson’s. Arts & Health. 2013;5: 103–119. doi: 10.1080/17533015.2012.745580 23805165 PMC3687249

[22] Volpe D, Signorini M, Marchetto A, Lynch T, Morris ME. A comparison of Irish set dancing and exercises for people with Parkinson’s disease: a phase II feasibility study. BMC geriatrics. 2013;13: 1–6. 23731986 10.1186/1471-2318-13-54 PMC3685562

[23] Bearss KA, Desouza JFX. Parkinson’s Disease Motor Symptom Progression Slowed with Multisensory Dance Learning over 3-Years: A Preliminary Longitudinal Investigation. Brain Sciences. 2021. doi: 10.3390/brainsci11070895 34356129 PMC8303681

[24] Kipnis D, Kruusamäe H, King M, Schreier AR, Quinn L, Shih H-JS. Dance interventions for individuals post-stroke—a scoping review. Topics in Stroke Rehabilitation. 2022;0: 1–18. doi: 10.1080/10749357.2022.2107469 35968809

[25] Ruiz-Muelle A, López-Rodríguez MM. Dance for People with Alzheimer’s Disease: A Systematic Review. Current Alzheimer Research. 2019;16: 919–933. doi: 10.2174/1567205016666190725151614 31345149

[26] Bek J, Arakaki AI, Lawrence A, Sullivan M, Ganapathy G, Poliakoff E. Dance and Parkinson’s: A review and exploration of the role of cognitive representations of action. Neuroscience and Biobehavioral Reviews. 2020;109. doi: 10.1016/j.neubiorev.2019.12.023 31846651

[27] Dhami P, Moreno S, DeSouza JFX. New framework for rehabilitation—fusion of cognitive and physical rehabilitation: the hope for dancing. Frontiers in Psychology. 2015;5. doi: 10.3389/fpsyg.2014.01478 25674066 PMC4309167

[28] Rehfeld K, Lüders A, Hökelmann A, Lessmann V, Kaufmann J, Brigadski T, et al. Dance training is superior to repetitive physical exercise in inducing brain plasticity in the elderly. PLoS ONE. 2018;13: e0196636. doi: 10.1371/journal.pone.0196636 29995884 PMC6040685

[29] Morrow-Howell N, Galucia N, Swinford E. Recovering from the COVID-19 Pandemic: A Focus on Older Adults. Journal of Aging and Social Policy. 2020;32: 526–535. doi: 10.1080/08959420.2020.1759758 32336225

[30] Wu B. Social isolation and loneliness among older adults in the context of COVID-19: a global challenge. Global Health Research and Policy. 2020;5: 1–3. doi: 10.1186/s41256-020-00154-3 32514427 PMC7272234

[31] Ellis TD, Earhart GM. Digital Therapeutics in Parkinson’s Disease: Practical Applications and Future Potential. Journal of Parkinson’s Disease. 2021;11: S95–S101. doi: 10.3233/JPD-202407 33646177 PMC8292155

[32] Quinn L, MacPherson C, Long K, Shah H. Promoting physical activity via telehealth in people with Parkinson disease: The path forward after the COVID-19 pandemic? Physical Therapy. 2020. doi: 10.1093/ptj/pzaa128 32734298 PMC7454884

[33] Sepúlveda-Loyola W, Rodríguez-Sánchez I, Pérez-Rodríguez P, Ganz F, Torralba R, Oliveira DV, et al. Impact of Social Isolation Due to COVID-19 on Health in Older People: Mental and Physical Effects and Recommendations. J Nutr Health Aging. 2020;24: 938–947. doi: 10.1007/s12603-020-1469-2 33155618 PMC7597423

[34] Moore RC, Hancock JT. Older Adults, Social Technologies, and the Coronavirus Pandemic: Challenges, Strengths, and Strategies for Support. Social Media + Society. 2020;6: 2056305120948162. doi: 10.1177/2056305120948162

[35] Chen AT, Ge S, Cho S, Teng AK, Chu F, Demiris G, et al. Reactions to COVID-19, information and technology use, and social connectedness among older adults with pre-frailty and frailty. Geriatric Nursing. 2021;42: 188–195. doi: 10.1016/j.gerinurse.2020.08.001 32863038 PMC7416746

[36] Schirinzi T, Di Lazzaro G, Salimei C, Cerroni R, Liguori C, Scalise S, et al. Physical Activity Changes and Correlate Effects in Patients with Parkinson’s Disease during COVID-19 Lockdown. Movement Disorders Clinical Practice. 2020. doi: 10.1002/mdc3.13026 32837960 PMC7404747

[37] Hülür G, Macdonald B. Rethinking social relationships in old age: Digitalization and the social lives of older adults. American Psychologist. 2020;75: 554–566. doi: 10.1037/amp0000604 32378949

[38] Silva PA. Are We Ready to Dance at Home?: A Review and Reflection of Available Technologies. Lecture Notes in Computer Science (including subseries Lecture Notes in Artificial Intelligence and Lecture Notes in Bioinformatics). Springer Verlag; 2019. pp. 216–231. doi: 10.1007/978-3-030-22015-0_17

[39] Shanahan J, Morris ME, Bhriain ON, Volpe D, Lynch T, Clifford AM. Dancing for Parkinson Disease: A Randomized Trial of Irish Set Dancing Compared With Usual Care. Archives of Physical Medicine and Rehabilitation. 2017. doi: 10.1016/j.apmr.2017.02.017 28336345

[40] Afshari M, Yang A, Bega D. Motivators and Barriers to Exercise in Parkinson’s Disease. Journal of Parkinsons Disease. 2017;7: 703–711. doi: 10.3233/JPD-171173 29103050

[41] Ellis T, Boudreau JK, DeAngelis TR, Brown LE, Cavanaugh JT, Earhart GM, et al. Barriers to exercise in people with Parkinson disease. Physical Therapy. 2013;93: 628–636. doi: 10.2522/ptj.20120279 23288910 PMC3641403

[42] Bek J, Groves M, Leventhal D, Poliakoff E. Dance at home for people with Parkinson’s during COVID-19 and beyond: Participation, perceptions, and prospects. Frontiers in Neurology. 2021. 10.3389/fneur.2021.678124 PMC8204717 34140925

[43] R Core Team 2021. In: R: A Language and Environment for Statistical Computing. 2021.

[44] Bates D, Maechler M, Bolker B, Walker S. Package"lme4". Journal of Statistical Software. 2015.

[45] Braun V, Clarke V. Using thematic analysis in psychology. Qualitative Research in Psychology. 2006. doi: 10.1191/1478088706qp063oa

[46] Carapellotti AM, Stevenson R, Doumas M. The efficacy of dance for improving motor impairments, non-motor symptoms, and quality of life in Parkinson’s disease: A systematic review and meta-analysis. PLoS ONE. 2020. doi: 10.1371/journal.pone.0236820 32756578 PMC7406058

[47] Morris ME, Slade SC, Wittwer JE, Blackberry I, Haines S, Hackney ME, et al. Online Dance Therapy for People With Parkinson’s Disease: Feasibility and Impact on Consumer Engagement. Neurorehabil Neural Repair. 2021;35: 1076–1087. doi: 10.1177/15459683211046254 34587834

[48] Ghanai K, Barnstaple RE, DeSouza JF. Virtually in synch: a pilot study on affective dimensions of dancing with Parkinson’s during COVID-19. Research in Dance Education. 2021: 1–15. doi: 10.1080/14647893.2021.2005560

[49] Bennett HB, Walter CS, Oholendt CK, Coleman KS, Vincenzo JL. Views of in-person and virtual group exercise before and during the pandemic in people with Parkinson disease. PM&R. 2022. doi: 10.1002/pmrj.12848 35596118 PMC10119971

[50] Bek J, Arakaki AI, Derbyshire-Fox F, Ganapathy G, Sullivan M, Poliakoff E. More Than Movement: Exploring Motor Simulation, Creativity, and Function in Co-developed Dance for Parkinson’s. Frontiers in Psychology. 2022;13: 326. doi: 10.3389/fpsyg.2022.731264 35295373 PMC8918650

[51] Hackney ME, Kantorovich S, Earhart GM. A study on the effects of argentine tango as a form of partnered dance for those with Parkinson Disease and the healthy elderly. American Journal of Dance Therapy. 2007;29: 109–127. doi: 10.1007/s10465-007-9039-2

[52] Fontanesi C, DeSouza JFX. Beauty That Moves: Dance for Parkinson’s Effects on Affect, Self-Efficacy, Gait Symmetry, and Dual Task Performance. Frontiers in Psychology. 2021;11: 3896–3896. doi: 10.3389/fpsyg.2020.600440 33613357 PMC7892443

[53] Blasing B, Calvo-Merino B, Cross ES, Jola C, Honisch J, Stevens CJ. Neurocognitive control in dance perception and performance. Acta Psychologica. 2012;139: 300–308. doi: 10.1016/j.actpsy.2011.12.005 22305351

[54] Hardwick RM, Caspers S, Eickhoff SB, Swinnen SP. Neural correlates of action: Comparing meta-analyses of imagery, observation, and execution. Neuroscience and Biobehavioral Reviews. 2018;94: 31–44. doi: 10.1016/j.neubiorev.2018.08.003 30098990

[55] Bek J, Holmes PS, Craig CE, Franklin ZC, Sullivan M, Webb J, et al. Action Imagery and Observation in Neurorehabilitation for Parkinson’s Disease (ACTION-PD): Development of a User-Informed Home Training Intervention to Improve Functional Hand Movements. Parkinson’s Disease. 2021: 1–14. doi: 10.1038/s41531-020-00149-4 PMC8324342 34336183

[56] Caligiore D, Mustile M, Spalletta G, Baldassarre G. Action observation and motor imagery for rehabilitation in Parkinson’s disease: A systematic review and an integrative hypothesis. Neuroscience and Biobehavioral Reviews. 2017;72: 210–222. doi: 10.1016/j.neubiorev.2016.11.005 27865800

[57] Abbruzzese G, Avanzino L, Marchese R, Pelosin E. Action Observation and Motor Imagery: Innovative Cognitive Tools in the Rehabilitation of Parkinson’s Disease. Parkinson’s Disease. 2015. doi: 10.1155/2015/124214 26495150 PMC4606219

[58] Temporiti F, Adamo P, Cavalli E, Gatti R. Efficacy and Characteristics of the Stimuli of Action Observation Therapy in Subjects With Parkinson’s Disease: A Systematic Review. Frontiers in Neurology. 2020;11: 808–808. doi: 10.3389/fneur.2020.00808 32903559 PMC7438447

[59] Harrison EC, McNeely ME, Earhart GM. The feasibility of singing to improve gait in Parkinson disease. Gait & Posture. 2017;53: 224–229. doi: 10.1016/j.gaitpost.2017.02.008 28226309 PMC5373799

[60] Irons JY, Hancox G, Vella-Burrows T, Han E-Y, Chong H-J, Sheffield D, et al. Group singing improves quality of life for people with Parkinson’s: an international study. Aging & Mental Health. 2021;25: 650–656. doi: 10.1080/13607863.2020.1720599 32020816

[61] Tamplin J, Morris ME, Marigliani C, Baker FA, Noffs G, Vogel AP. ParkinSong: Outcomes of a 12-Month Controlled Trial of Therapeutic Singing Groups in Parkinson’s Disease. Journal of Parkinson’s Disease. 2020;10: 1217–1230. doi: 10.3233/JPD-191838 32538865

[62] Tamplin J, Morris ME, Baker FA, Sousa TV, Haines S, Dunn S, et al. ParkinSong Online: protocol for a telehealth feasibility study of therapeutic group singing for people with Parkinson’s disease. BMJ Open. 2021;11: e058953. doi: 10.1136/bmjopen-2021-058953 34930750 PMC8689189

[63] Hackney ME, Earhart GM. Effects of dance on gait and balance in Parkinsons disease: A comparison of partnered and nonpartnered dance movement. Neurorehabilitation and Neural Repair. 2010;24: 384–392. doi: 10.1177/1545968309353329 20008820 PMC2900796

[64] Kunkel D, Robison J, Fitton C, Hulbert S, Roberts L, Wiles R, et al. It takes two: the influence of dance partners on the perceived enjoyment and benefits during participation in partnered ballroom dance classes for people with Parkinson’s. Disability and Rehabilitation. 2018;40. doi: 10.1080/09638288.2017.1323029 28482703

[65] Ashworth NL, Chad KE, Harrison EL, Reeder BA, Marshall SC. Home versus center based physical activity programs in older adults. Cochrane Database of Systematic Reviews. 2005. doi: 10.1002/14651858.CD004017.pub2 15674925 PMC6464851

[66] Kelly MP, Leventhal D. Dance as Lifeline: Transforming Means for Engagement and Connection in Times of Social Isolation. Health Promotion Practice. 2021;22: 64S–69S. doi: 10.1177/1524839921996332 33942644

[67] Lee N-Y, Lee D-K, Song H-S. Effect of virtual reality dance exercise on the balance, activities of daily living, and depressive disorder status of Parkinson’s disease patients. Journal of Physical Therapy Science. 2015. doi: 10.1589/jpts.27.145 25642060 PMC4305547

