# Supplementary material for: Moving online: Experiences and potential benefits of digital dance for older adults and people with Parkinson’s disease
Source: PLoS One. 2022 Nov 18;17(11):e0277645. doi: 10.1371/journal.pone.0277645 (PMC9674152; doi:10.1371/journal.pone.0277645)
Supplement: S1 File — (PDF) [file pone.0277645.s001.pdf]

S1: Survey questions

Dancing at home survey

Thank you for agreeing to participate in this study. Please complete the questions below. If you are not able to complete all sections, any responses you do provide will still be very helpful.

**1**	**What is your age?**	
**2**	**What is your gender?**	
	Female	
	Male	
	I do not identify as male or female	
	Prefer not to answer	
**3**	**What is your location (city/town; country)?**	
**4**	**Have you been diagnosed with a neurological condition?**<br>Please select all that apply:	
	None	
	Parkinson's	
	Multiple Sclerosis	
	Stroke	
	Other (please specify)	
**4(a)**	If yes to any of the above: Approximately how long ago were you diagnosed?	
**4(b)**	If yes to any of the above: How would you rate the overall severity of your condition/symptoms?	
	Mild	
	Moderate	
	Severe	
**5**	**Do you have any other health conditions that affect your movement or mobility?**	
	Yes	
	No	

**6**	**Do you use any at-home dance resources (e.g., live-streamed classes, online videos, DVDs)?**		
	Yes		***Please go to Q7**
	No		***Please go to Q8**
**7**	**Which resources have you used?**		
	Live streamed classes		
	Interactive online classes (e.g., using video-conferencing platforms)		
	Pre-recorded online classes		
	DVD		
	Other (please specify)		
	**Please state programs you have used if possible (e.g., Silver Swans, Dance for PD)**		
**8**	**If "no" to Q6, why not?** *Please select all that apply:*		
	I don't have the time		
	I'm not interested		
	I don't know how to find/use them		
	I don't have access to the internet/software needed		
	I don't feel motivated enough		
	I don't think they will benefit me		
	Other (please specify)		
**9**	**Which features are *or would be* important to you when choosing an at-home class?** *Please select all that apply:*		
	Type of media/technology platform		
	Low cost		
	Free classes only		
	Reputation/brand		
	Recommendation		
	Familiar program/instructor		
	Dance style (e.g., ballet, modern, mixed)		
	Opportunities for social connection (e.g., online 'chat' time around the class)		

	Difficulty level	
	Scheduled classes (specific/regular time)	
	Other (please specify)	

***If you do <u>not</u> use any at-home dance resources, please now go to Q23**

**10**	**If using different types of at-home resources (e.g., live, pre-recorded), which do you prefer and why? (optional)**	
**11**	**How long have you been using at-home dance resources?**	
**12**	**Why did you start using at-home dance resources?** *Please select all that apply:*	
	In-person classes suspended/no longer available	
	No in-person class near to me	
	Prefer to dance at home	
	Wanted additional practice alongside in-person classes	
	Recommended by a friend/relative/health professional	
**13**	**Did you start using at-home dance resources because classes were suspended due to COVID-19 restrictions?**	
	Yes	
	No	
	I was already using at-home dance resources	
	I was already considering trying at-home dance resources	
**14**	**How often are you using at-home dance resources?**	
	Once a week	
	Twice a week	
	Less frequent (please specify)	
	More frequent (please specify)	
**15**	**Do you practice alone or with a family member/partner/friend?**	
	Alone	
	With family member/partner/friend - by choice	
	With family member/partner/friend - I need additional support	

**16**	**Have you experienced any difficulties with these resources?**<br>*Please select all that apply:*	
	No problems	
	Connectivity/network problems (e.g., freezing, intermittent access)	
	Setting up or using software (e.g., video-conferencing app)	
	Image quality	
	Sound quality	
	Other (please specify)	
**17**	**We would like to know a bit more about your experience of participating in dance at home.**<br>**Have you noticed that you have done any of the following during your practice…?**<br>*Please select all that apply:*	
	Watching the instructor closely	
	Vocalising the movements (talking through what I am doing)	
	Singing	
	Counting (e.g., to help with timing)	
	Imagining how the movements would look if I did them	
	Imagining how the movements would feel if I did them	
	Imagining moving like something else (an animal, a tree, waves on the ocean, falling leaves etc.)	
	Playing my own music in the background	
	Other (please specify)	
**18**	**If using pre-recorded videos, do you prefer to join in with the instructor, or watch and then practice (e.g., pausing the video to try out the movements)?**	
	Practice in time with the instructor	
	Watch and then practice	
	Practice in time with instructor then pause and practice again	
	Watch and just join in the parts I am able to do	
	I don't use pre-recorded videos	
**19**	**Have you noticed any benefits of your home practice?**<br>*Please select all that apply:*	
	Moving more easily while dancing	

	Moving more easily in general	
	Performing everyday activities or household tasks more easily/effectively	
	Improved balance	
	Improved posture	
	Improved rhythm	
	Improved concentration	
	Improved mood	
	Reduced stress/anxiety	
	Increased energy	
	Increased confidence	
	Sleeping better	
	No benefits	
	Other (please specify)	
**20**	**What are the advantages of at-home dance?** *Please select all that apply:*	
	Doesn't require travel	
	Flexible timing allows me to fit practice into my day/week	
	Ease of access motivates me to participate more often	
	Allows me to maintain privacy	
	I can go at my own pace	
	I can express myself without worrying what others think	
	Other (please specify)	
**21**	**What are the disadvantages of at-home dance?** *Please select all that apply:*	
	Reduced motivation to participate without a fixed routine (e.g., weekly scheduled class)	
	Difficulties in accessing/using technology	
	I don't have a suitable space at home	
	Difficulty in finding quiet time to practice	
	Lack of one-to-one support/tuition	
	Lack of social interaction	
	Other (please specify)	

**22**	**Do you have any suggestions for how at-home dance resources could be improved? (optional)**		
**23**	**Have you previously attended in-person classes?**		
	Yes		***Please go to Q24**
	No		***Please go to Q26**
**24**	**If "yes" please state approximately how frequently and how long you have been attending**		
**25**	**What do you miss about in-person classes?** *Please select all that apply:*		
	Interaction with the instructor		
	Interaction with others		
	Support/encouragement		
	Live music		
	Social activities before/after class		
	Other (please specify)		
**26**	**When in-person classes resume, what would you like to do?**		
	Attend classes only		
	Continue with home practice only		
	Both attend classes and continue with home practice		

**27**	**Would you be interested in receiving more information that might help you to get the most out of your dance practice (e.g., particular skills that dance may develop)?**		
		**Yes**	**No**
	Written information		
	Educational video		
**28**	**Would you be interested in trying the following in the future?**		
		**Yes**	**No**
	Practicing dance movements using an app (program on a tablet computer or smartphone)		
	Virtual reality dance (wearing a headset that immerses you in a "virtual dance class")		

**29**	**Are you using any other at-home activity resources (e.g., yoga, exercise classes)?**	
	Yes	
	No	
**30**	**If "yes", please tell us which other resources you are using (optional)**	
**31**	**Are you using any online resources/platforms to socialise and stay connected with others?**	
	Yes	
	No	
**32**	**If "yes", please tell us which resources you are using (optional)**	
**33**	**Is there anything else you would like to tell us about your experience of dance at home resources? (optional)**	
