# Supplementary material for: Moving online: Experiences and potential benefits of digital dance for older adults and people with Parkinson’s disease
Source: PLoS One. 2022 Nov 18;17(11):e0277645. doi: 10.1371/journal.pone.0277645 (PMC9674152; doi:10.1371/journal.pone.0277645)
Supplement: S2 File — (PDF) [file pone.0277645.s002.pdf]

S2: Themes generated from comments of older adults (OA) and individuals with PD (PD) in response to open survey questions

(1) Maintaining connection and well-being

This theme reinforced findings from the fixed-choice questions, indicating that participants missed the social connection from in-person classes.

> *"Nothing replaces actually being in the dance studio with a teacher and other dancers" [OA]*
>
> *"Although I myself am enjoying them at present, I still prefer real life classes, with all their interaction" [PD]*
>
> *"I find the online classes frustrating (though wonderful) in a way…we are missing the expressions of the other students, the subtleties of personal expression, the smiles, the nods, the ability to work in pairs or groups and separate out to create" [OA]*

However, a sense of community could be found through connecting with others online.

> *"You feel connected to the rest of the group even if it's done remotely" [OA]*
>
> *"…feel I am part of something even though no-one else is there!" [PD]*

Being able to access at-home dance programs supported participants in maintaining their physical and mental health during COVID-19 restrictions.

> *"They do enable us to…protect our state of mind and maintain a level of fitness that would otherwise diminish" [OA]*
>
> *"The online Zoom class the teacher offered and the discovery of online pre-recorded classes has helped with coping with the lockdown restrictions on my life" [OA]*
>
> *"I feel as though I have met new friends. What could have been better during this period of lockdown and global pandemic. I do think it is of paramount importance that people of my age continue to do the things they love, to keep them fit and healthy and in a good place mentally." [OA]*
>
> *"I am really enjoying dance at home and feel much better physically and mentally after a class. It is especially beneficial at this time, when there are so many restrictions." [PD]*

One participant also noted that practising at home helped to maintain confidence for returning to regular group sessions.

> *"It also gives me confidence in my ability when I go to live classes" [OA]*

Many respondents simply expressed gratitude for the adaptations made by instructors and organisations that provided the opportunity to continue dancing.

> *"I feel extremely fortunate to have been able to follow classes online" [OA]*
>
> *"They have been a life saver especially during this troubling time" [PD]*

(2) Advantages and opportunities in online participation

This theme highlighted advantages of at-home participation, such as the convenience of not having to travel, the capacity to dance more frequently, and the provision of a safe space for movement and expression.

"In several ways I have found this preferable to actually being in class (not having to travel...)" [OA]

"Please continue after COVID-19. No traveling to class huge benefit!!!" [PD]

"I have been practising regularly and find my balance in particular is very much improved… I must confess that before lockdown the only time I danced was in class once a week with no practice in between." [OA]

"The number of times each week it allows me to dance is terrific. I love dancing every day - sometimes morning and afternoon." [PD]

"The online experience of classes enables me to choose (having tried several different ones) which one I feel I am able to do that day and at a time convenient to me." [OA]

"Allows people who are physically restricted to move freely and use their creativity and imagination without being judged" [PD]

The desire to continue with online classes was also echoed.

"I have enjoyed the dance experiences and intend to continue in class and online in the future" [OA]

"Please keep this available as I live in a small town with 450 people and no activity like this offered" [PD]

"They are a tremendous help during the pandemic, but I shall continue using them afterwards" [PD]

"I also believe it would continue to be useful in the future for those who are unable to travel to ordinary live classes, and for those who are keen to dance as much as possible" [PD]

Online participation led to new experiences and opportunities for some participants, such as the possibility of joining classes in remote locations and meeting new people online. Others had been able to try new classes or dance styles by searching online, or even discovered dance for the first time.

"I have also enjoyed some online classes found on YouTube which were taught in different ways" [OA]

"I also like trying out new classes and if I like the video, I add them on to my workout selection" [PD]

"COVID19 lockdown led me to join an online community of over 50s dancers where I was made welcome. I would never have met these dancers under normal circumstances because they are based in Yorkshire over 100 miles away from my home." [OA]

"I also like meeting dancers from all over the world. If not virtual, I would not be able to attend. A gift!" [PD]

"…the interaction with people from across the world was fascinating and motivating" [PD]

It was also noted that the learning of new technology skills had been accelerated during the pandemic, although there may have been initial challenges.

"It has made me feel more confident using video and computer technology" [OA]

"They started a Zoom class early in lockdown but I got put off when they were getting it going, and didn't realise I could highlight one dancer to follow and was trying to follow one person when I was still in gallery mode!" [PD]

Some individuals had found ways of using the digital format to create choreographies in online classes.

"It has opened up new creative pathways to explore and introduced an interesting visual perception of how other members of the group move and react to the process of creating choreography online." [OA]

(3) Effectiveness of engagement, teaching, and learning

This theme encapsulated both positive and negative aspects of at-home participation, and elements that respondents considered important for optimising or improving the experience.

It was noted that it could be difficult to maintain motivation, although scheduled classes helped to encourage regular participation.

> *"It's very convenient but you have to be self motivated" [PD]*

> *"I find having a regular time slot enables me to plan for it" [PD]*

> *"It is very difficult to keep myself motivated. It is the classes that I know I will return to after lockdown at some point that keep me going." [OA]*

> *"It was good to be able to keep the connection with the weekly class as it helps motivate a structure in the day at a specific time." [OA]*

It was also suggested that smaller classes with a regular group would be preferable.

> *"Teachers should maybe keep a class small and there should be a registration so you are in an actual group like a real class without so much come and go of the students" [OA]*

> *"Smaller classes so some personal feedback possible" [OA]*

The importance of having instructors who were experienced in working with a particular group and sensitive to the needs of participants was highlighted. Familiarity with the instructor also appeared to increase motivation and engagement.

> *"Our teacher takes into consideration actions we may be unable to do at our age. She is good at breaking down each routine into smaller 'bites'." [OA]*

> *"I like the contact with my regular teacher and understand her way of teaching" [OA]*

> *"We have an excellent teacher who is very good at explaining the steps. This makes our on line classes very enjoyable. If our teacher was not as good, this might be a problem." [OA]*

> *"The teacher … uses music as background sound but does not coordinate the rhythm and the movements or consider the effect of the tempo on the movement. My movement is more fluid if I have appropriate external cues." [PD]*

> *"I love the enthusiasm of the teachers" [PD]*

While the above points could apply equally to in-person and digital classes, other feedback indicated aspects more specific to the home-based context. For example, clear visibility of the movements was noted to be important to facilitate learning.

> *"I have difficulty following in mirror image, muddled with rights and lefts" [OA]*

> *"Demonstrations of the steps using more than one camera so we can see different angles" [OA]*

> *"One thing we really value in the at-home dance resources is when instructors are very visible and both describing and doing the movements when showing us a new segment" [PD]*

Another limitation was that the home environment could be restrictive or unsuitably equipped for dance.

> *"It would be great if it were possible to more easily purchase supplies for use at home -- e.g., we have a carpeted living room, and that is terrible for ballet. A suitable mat that could be rolled up and kept out of the way" [OA]*

"The space I have at home is not sufficient to do more than a barre and limited exercises in the centre" [OA]

"I find space restricts me - I like to really MOVE and travel while dancing" [PD]

Issues concerning accessibility were highlighted, such as the need to improve technical knowledge and the availability of guidance on how to find suitable resources.

"Training for both instructors and users on how best to use videoconferencing" [PD]

"Providing a directory or central portal where resources, classes etc. could be located" [OA]

Other comments indicated the need for greater diversity in provision, in order to facilitate participation across different locations, languages, and cultures.

"Could consider live participation and schedule more classes for other time zones" [PD]

"More classes in other languages… I often think about all the other people that may not participate because of language barriers. In general too, I think the at-home resources need to increase advertising for racial and ethnic minorities because it's a very homogeneous group that participates." [PD]

<u>(4) Different preferences</u>

It was evident that participants varied in their preferences when engaging with at-home dance. While many preferred live classes, others valued the flexibility of having recorded sessions.

"After watching and preparing the pre-recorded exercises I found it still very important to get instructions on which mistakes to avoid and personalised feedback to correct mistakes during the live sessions. Simply watching somebody who can do it well is not enough to learn how it feels to do it right." [OA]

"The interactive sessions are great fun, the pre-recorded sessions I have greater difficulty. I like both" [OA]

"I wish there were more opportunities to access more than one weekly interactive class" [PD]

"I would like to be able to access classes in a library, to be able to repeat them if wished" [PD]

Some participants wanted to be able to participate in a complete and continuous session, while others preferred to have exercises broken down into manageable segments.

"I would like a continuous 30-40 minute workout so I don't need to click on each exercise" [OA]

"Thinking of prerecorded videos only, it would be ideal to have starter videos that break down the steps" [PD]

Detailed verbal descriptions of the exercises were appreciated by some participants, but others found long explanations to be off-putting and to disrupt the flow of the class.

"One thing we really value in the at-home dance resources is when instructors are very visible and both describing and doing the movements when showing us a new segment" [PD]

"I feel that an edited version (to exclude the instructional dialogue) would be perfect for me" [OA]

"Some spend too much time on instruction which makes it hard to maintain momentum" [PD]
